# Translating and validating a Japanese version of the instrument for patient assessment of medical professionalism (J-IPAMP): a cross-sectional survey

**DOI:** 10.1186/s12909-022-03699-8

**Published:** 2022-08-23

**Authors:** Hirohisa Fujikawa, Daisuke Son, Takuya Aoki, Kayo Kondo, Yousuke Takemura, Minoru Saito, Naoko Den, Masato Eto

**Affiliations:** 1 Department of Medical Education Studies, International Research Center for Medical Education, Graduate School of Medicine, The University of Tokyo, 7-3-1 Hongo, Bunkyo-ku, Tokyo, 113-0033 Japan; 2 Department of Community-Based Family Medicine, Faculty of Medicine, Tottori University, Yonago, Tottori, Japan; 3 Division of Clinical Epidemiology, Research Center for Medical Sciences, The Jikei University School of Medicine, Minato-ku, Tokyo, Japan; 4 Section of Clinical Epidemiology, Department of Community Medicine, Graduate School of Medicine, Kyoto University, Sakyo-ku, Kyoto, Japan; 5 School of Modern Languages and Cultures, Durham University, Durham, UK; 6 Department of General Medicine, Graduate School of Medical Science, Tokyo Women’s Medical University, Shinjuku-ku, Tokyo, Japan; 7 Department of Internal Medicine, Suwa Central Hospital, Chino, Nagano, Japan; 8 Department of Internal Medicine, Oji Seikyo Hospital, Tokyo Hokuto Health Co-Operative, Kita-ku, Tokyo, Japan

**Keywords:** Medical professionalism, Reliability, Validity, Scale development

## Abstract

**Background:**

Although there are many tools to assess medical professionalism, they rarely address patients’ perspectives. The instrument for patient assessment of medical professionalism (IPAMP) comprises 11 items and has been established and validated as a valuable tool for assessing trainees’ professionalism from the patient’s perspective. However, there is no instrument to assess professionalism from the patient’s perspective in Japan. The purpose of the present study was to develop a Japanese version of the IPAMP (J-IPAMP) and test its validity and reliability.

**Methods:**

We conducted a cross-sectional survey to examine the reliability and validity of the J-IPAMP in two hospitals (one each in an urban and rural area) in Japan. Receptionists or surveyors distributed the anonymous questionnaire to 276 inpatients; all participants were aged above 20 years and assigned to medical trainees. We evaluated its structural and criterion-related validity, as well as internal consistency reliability.

**Results:**

Data of 235 (85.1%) patients were analyzed. Using the split-half validation technique, we performed an exploratory factor analysis (EFA) along with a confirmatory factor analysis (CFA). The EFA showed a one-factor solution. Then, to compare the model fitness between two models (the two-factor model from the original English version vs. unidimensional model suggested by the EFA), the CFA was performed. The CFA showed that almost all of the fit indices met their respective criteria and were approximately the same for the two models. Thus, we adopted a single-factor model. The Pearson correlation coefficients between the total J-IPAMP scores and the global ratings were 0.738, indicating adequate criterion-related validity. The Cronbach’s alpha of the 11 items of the instrument was 0.96 (95% confidence interval: 0.96–0.97) and the omega value was 0.96, demonstrating acceptable internal consistency reliability.

**Conclusions:**

We developed the Japanese version of the IPAMP. Its validity and reliability were verified through analysis. This instrument can be utilized for professionalism education in the postgraduate training setting.

## Background

In recent years, the importance of medical professionalism has been increasingly highlighted by scholars [1–3]. Nevertheless, it remains one of the most intangible and challenging concepts in medical education [1]. In 1999, it was listed as one of the six general competencies that physicians-in-training must develop, with several foundations in Europe and the United States collaborating to launch the Medical Professionalism Project in the same year. Afterwards, in 2002, the Physician’s Charter on Medical Professionalism was published [4, 5], which is now endorsed by more than 100 professional associations worldwide [6]. Thus, in the field of medical education, medical professionalism has received growing attention over the years.

The assessment of medical professionalism is important because it: (1) promotes learning, (2) facilitates the formation of a professional identity, (3) is vital in the quality improvement cycle, and (4) is crucial to identifying and remediating learners whose performance may endanger patients [7–9]. Although there are many tools to assess medical professionalism [10, 11], patients’ perspectives regarding the assessment of such professionalism have rarely been addressed [12]. Meanwhile, in other areas of medicine, such as patient safety and quality improvement, great strides have been made to incorporate patients’ perspectives, such as by assessing patient feedback [13]. Because patients are the key stakeholders for medical professionals [14], research on medical professionalism assessment using patients’ lived experiences is a matter of high priority [12, 15]. Wilkinson et al., in a recent systematic review aimed at producing a blueprint to assess medical professionalism [14], described the following specific physician behaviors that could be appropriately assessed by patients: respecting the diversity of patients, showing politeness and patience, having an empathetic and compassionate attitude, and engaging with patients in shared decision making [14]. As these behaviors are difficult to evaluate in official assessment settings, patients’ opinions regarding these behaviors are crucial in that they may complement other sources of information (e.g., paper-based test, simulation, and global view of supervisor).

In 2020, Ratelle et al. developed the Instrument for Patient Assessment of Medical Professionalism (IPAMP), which can be used to rate internal medicine trainees’ professionalism [16]. The assessment method was adapted from Dine et al. and the American Board of Internal Medicine [17–19], which were both well-validated for reliability and validity analysis. Ratelle et al. iteratively refined and validated the instrument. In Japan, assessing the medical professionalism of trainees is very important and a matter of great interest among researchers in this field [20]. However, no tools are available for assessing trainees’ professionalism from the patients’ perspectives in Japan. Therefore, we developed the Japanese version of the IPAMP (J-IPAMP) and examined its validity and reliability in a multicentered, cross-sectional study.

## Methods

### Study design, setting, and participants

The IPAMP was subjected to cross-cultural translation and adaptation into Japanese. We conducted a cross-sectional survey to examine its psychometric properties. We contacted two postgraduate clinical training hospitals (Oji Seikyo Hospital and Suwa Central Hospital) in Japan, and both agreed to cooperate. Oji Seikyo Hospital is located in an urban area and has 159 beds, whereas Suwa Central Hospital is in a rural area and has 360 beds. From September 2021 to March 2022, we distributed an anonymous self-administered version of the J-IPAMP to potential participants. The eligibility criteria for patients were: aged 20 years and above, admitted to one of these two hospitals, and assigned to clinical trainees (postgraduate years 1–5) during the survey period. All voluntarily agreed to join this study beforehand. Patients who were expected to find it difficult to answer the paper-based questionnaire on their own because of severe physical (e.g., fracture of the dominant arm) or mental (e.g., severe dementia) disorders, based on hospitalization observations by hospital staff, were excluded from the study. The questionnaires were delivered by the receptionists or surveyors at each institution. The respondents filled out the questionnaire, put it in an envelope, and dropped it in the collection box at each institution.

### Translation process

The original IPAMP has 11 items, each of which is rated on a 5-point Likert scale (1 = poor, 2 = fair, 3 = good, 4 = very good, and 5 = excellent). Factor analysis showed a two-factor model comprising “involvement and respect” (Q5, Q6, and Q8–Q11) and “compassion and rapport” (Q1–Q4 and Q7) [16].

After obtaining permission from the original author, we translated the IPAMP into Japanese following an international guideline for the cross-cultural adaptation process [21]. First, three forward translations from English to Japanese were conducted independently by three translators (HF, DS, and KK). All three were fluent in English, familiar with the cultures of healthcare in which both languages are used, and had professional experience translating questionnaires in the field of medical education [22]. Second, the translators worked together to synthesize and refine the Japanese translations (Version 1). Third, we requested professional bilingual translators who had no prior knowledge of the questionnaire to back translate Version 1 into English. We compared the back-translated version with the original English version, proofread it, and produced Version 2. Fourth, we asked an expert in medical professionalism (YT) to review Version 2 and modified it based on the feedback (Version 3). Fifth, we asked the original author to review Version 3 to ensure that there were no problems with the translation process. Finally, from July to August 2021, a pilot test was conducted on 11 inpatients at Suwa Central Hospital. The author (HF) interviewed the inpatients on whether Version 3 was intelligible and understood as intended. As the pilot test revealed that there were no problematic items in the translation processes, Version 3 was considered final. The tool’s face and content validity were ensured by all authors.

### Statistical analysis

The structural validity of the J-IPAMP was tested through both exploratory factor analysis (EFA) and confirmatory factor analysis (CFA). We applied the split-half validation approach and randomly split the sample into two independent groups. As this study sought to develop a scale that is optimized for the Japanese healthcare context, we decided to perform the EFA first.

Before the EFA, the Kaiser–Meyer–Olkin (KMO) measure of sampling adequacy and Bartlett’s test of sphericity were performed to assess the suitability of the data for performing EFA. A KMO value greater than 0.60 [23] and a significant Bartlett’s test (*p* < 0.05) indicates suitability for EFA. EFA was conducted on half of the dataset using the maximum likelihood with promax rotation method. For factor extraction, Kaiser-Guttman Criterion (Eigenvalues greater than 1) and parallel analysis were employed [23]. A cut-off value of 0.40 was adopted for factor loadings.

We conducted CFA using the maximum likelihood estimation approaches on the other half of the data to assess the suitability of the original two-factor model and identify an alternative model. The model fitness of the data was determined by calculating goodness-of-fit indices. We employed the following criteria to assess the model fitness: comparative fit index (CFI) close to 0.90 or higher, Tucker–Lewis index (TLI) close to 0.90 or higher, root mean square error of approximation (RMSEA) close to 0.08 or below, and standardized root mean square residual (SRMR) close to 0.08 or below [23–25].

We used the J-IPAMP total scores and the global rating to examine criterion-related validity. The question for the global rating was as follows: “Using any number from 0 to 10, where 0 is the worst doctor possible and 10 is the best doctor possible, what number would you use to rate this doctor as a professional doctor?” Criterion-related validity was assessed using Pearson correlation coefficients between the J-IPAMP total scores and the global rating. Correlation coefficients were considered meaningful if they were above 0.30 [26]. Finally, we utilized Cronbach’s alpha and omega coefficients to examine the internal consistency reliability of the scale. A value of above 0.70 for both coefficients is considered satisfactory reliability [27, 28]. We chose a complete case analysis method because of the small amount of missing data. We analyzed the data using R version 4.2.1 (R Foundation for Statistical Computing, Vienna, Austria; www.R-project.org). We used psych version 2.2.5 and GPArotation version 2022.4–1 to perform EFA and lavaan version 0.6–12 and semPlot version 1.1.6 to conduct CFA [29–32].

### Ethical considerations

All participants gave their oral consent before the study began. We obtained ethics approval from the Institutional Review Board of the University of Tokyo (2021074NI).

## Results

**Fig. 1:**
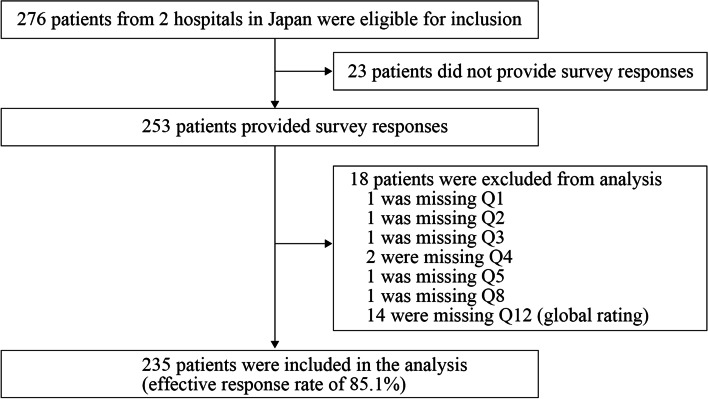
Flowchart of participants in a study of the translating and validating a Japanese version of the instrument for patient assessment of medical professionalism

Of the 276 eligible participants, 253 (91.7%) completed the questionnaire. We excluded 18 responses because of missing data. Thus, we included 235 (85.1%) patients in the analysis (Fig. 1). Table 1 summarizes the participants’ characteristics, and Table 2 shows the participants’ responses to each item of the J-IPAMP.

**Table 1:** Participants’ characteristics

Characteristic	N (%)
Gender	
Female	72 (30.6)
Male	161 (68.5)
Unanswered	1 (0.4)
Data missing	1 (0.4)
Age (years)	
20–24	7 (3.0)
25–34	6 (2.6)
35–44	9 (3.8)
45–54	29 (12.3)
55–64	33 (14.0)
65–74	74 (31.5)
75 or more	77 (32.8)
Education	
Less than high school	32 (13.6)
High school	118 (50.2)
Junior college	35 (14.9)
More than or equal to college	46 (19.6)
Data missing	4 (1.7)
Department	
Internal medicine	148 (63.0)
General medicine	39 (16.6)
Orthopedics	31 (13.2)
Surgery	7 (3.0)
Otorhinolaryngology	6 (2.6)
Data missing	4 (1.7)
Duration of hospitalization (days)	
1–10	145 (61.7)
11–20	55 (23.4)
21–30	14 (6.0)
31–40	7 (3.0)
41–50	4 (1.7)
51–60	2 (0.9)
> 60	6 (2.6)
Data missing	2 (0.9)

**Table 2:** Response to the Japanese version of the instrument for patient assessment of medical professionalism (*N* = 235): Number (%)

Items (as in original version)	Poor	Fair	Good	Very good	Excellent
Q1. How is this doctor at letting you tell your story; listening carefully; asking thoughtful questions; not interrupting you while you’re talking?	0 (0)	20 (8.5)	55 (23.4)	82 (34.9)	78 (33.2)
Q2. How is this doctor at showing interest in you as a person; not acting bored or ignoring what you have to say?	1 (0.4)	16 (6.8)	55 (23.4)	73 (31.1)	90 (38.3)
Q3. How is this doctor in treating you like you’re on the same level; never “talking down” to you or treating you like a child?	0 (0)	10 (4.3)	46 (19.6)	75 (31.9)	104 (44.3)
Q4. How is this doctor in greeting you warmly; calling you by the name you prefer; being friendly, never crabby or rude?	1 (0.4)	13 (5.5)	44 (18.7)	72 (30.6)	105 (44.7)
Q5. How is this doctor at telling you everything; being truthful, upfront, and frank; not keeping things from you that you should know?	1 (0.4)	15 (6.4)	55 (23.4)	71 (30.2)	93 (39.6)
Q6. How is this doctor at warning you during the physical exam about what he/she is going to do and why; telling you what he/she finds?	4 (1.7)	29 (12.3)	50 (21.3)	69 (29.4)	83 (35.3)
Q7. How is this doctor at using words you can understand when explaining your problems and treatment; explaining any technical medical terms in plain language?	2 (0.9)	19 (8.1)	55 (23.4)	74 (31.5)	85 (36.2)
Q8. How is this doctor at respecting your thoughts and beliefs; putting himself/herself “in your shoes”?	3 (1.3)	24 (10.2)	54 (23.0)	73 (31.1)	81 (34.5)
Q9. How is this doctor at explaining what you need to know about your problems, how and why they occurred, and what to expect next?	4 (1.7)	33 (14.0)	54 (23.0)	68 (28.9)	76 (32.3)
Q10. How is this doctor at discussing options with you; asking your opinion; offering choices and letting you help decide what to do; asking what you think before telling you what to do?	6 (2.6)	29 (12.3)	61 (26.0)	71 (30.2)	68 (28.9)
Q11. How well did this doctor keep you informed about your plan of care; notifying you of upcoming tests and treatments?	8 (3.4)	28 (11.9)	51 (21.7)	71 (30.2)	77 (32.8)
**Global rating**	**0–2**	**3–4**	**5–6**	**7–8**	**9–10**
Using any number from 0 to 10, where 0 is the worst doctor possible and 10 is the best doctor possible, what number would you use to rate this doctor as a professional doctor?	1 (0.4)	5 (2.1)	33 (14.0)	93 (39.6)	103 (43.8)

### Psychometric analyses

First, we performed EFA on 117 participants to assess the structural validity of the J-IPAMP. The KMO value was 0.945, which exceeded the 0.60 threshold. Bartlett’s test was significant with *p* < 0.001 (χ^2^ = 1432.036, df = 55). These results indicated that the data were appropriate for EFA. Table 3 presents the results of the EFA. Both the Kaiser-Guttman Criterion and parallel analysis showed that a one-factor structure for all 11 items fit the data best, and all factor loadings were more than 0.40. The one-factor model explained 73.7% of the variance.

**Table 3:** Factor loadings in exploratory factor analysis for the Japanese version of the instrument for patient assessment of medical professionalism

Items (as in original version)	Factor 1
Q1. How is this doctor at letting you tell your story; listening carefully; asking thoughtful questions; not interrupting you while you’re talking?	0.870
Q2. How is this doctor at showing interest in you as a person; not acting bored or ignoring what you have to say?	0.883
Q3. How is this doctor in treating you like you’re on the same level; never “talking down” to you or treating you like a child?	0.790
Q4. How is this doctor in greeting you warmly; calling you by the name you prefer; being friendly, never crabby or rude?	0.814
Q5. How is this doctor at telling you everything; being truthful, upfront, and frank; not keeping things from you that you should know?	0.857
Q6. How is this doctor at warning you during the physical exam about what he/she is going to do and why; telling you what he/she finds?	0.889
Q7. How is this doctor at using words you can understand when explaining your problems and treatment; explaining any technical medical terms in plain language?	0.869
Q8. How is this doctor at respecting your thoughts and beliefs; putting himself/herself “in your shoes”?	0.894
Q9. How is this doctor at explaining what you need to know about your problems, how and why they occurred, and what to expect next?	0.855
Q10. How is this doctor at discussing options with you; asking your opinion; offering choices and letting you help decide what to do; asking what you think before telling you what to do?	0.865
Q11. How well did this doctor keep you informed about your plan of care; notifying you of upcoming tests and treatments?	0.854
	**Value**
Eigenvalue	8.109
Percentage variance explained	73.707

**Fig. 2:**
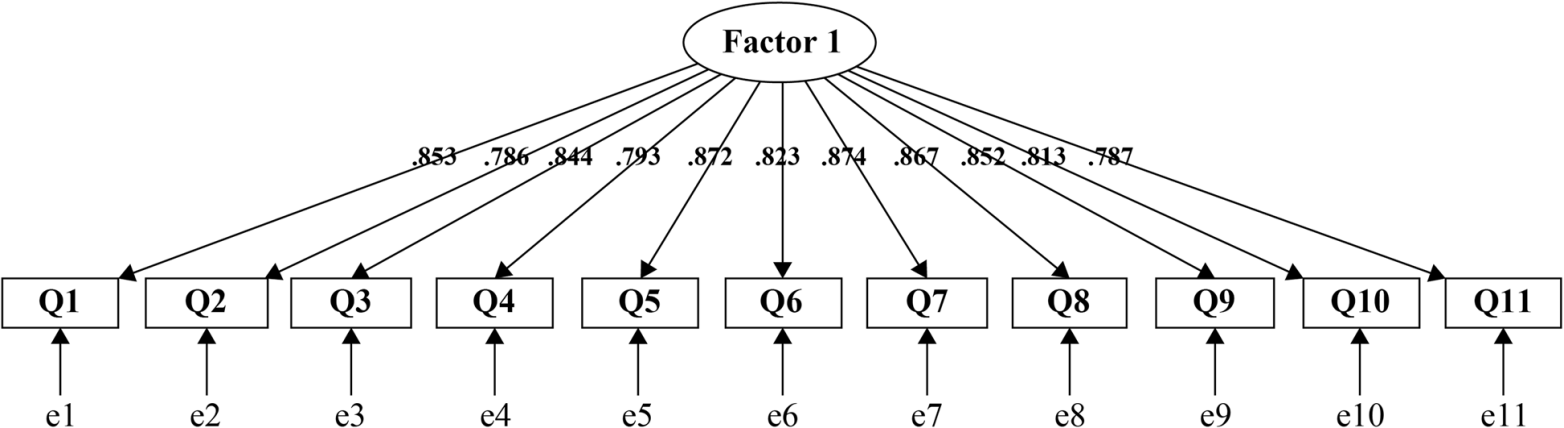
Path diagram for the confirmatory factor analysis of the Japanese version of the instrument for patient assessment of medical professionalism (the final one-factor model). The ellipse is a latent variable (factor). The rectangles are observed variables (items). The values on the arrows are standardized factor loadings

We then conducted CFA on the remaining 118 participants for the following two models: the two-factor model from the original English version and a unidimensional model suggested by EFA. The goodness-of-fit results are presented in Table 4. All the fit indices, except RMSEA, met their respective criteria and were approximately the same for both models. Considering the results of the factor analysis, we decided to adopt a one-factor model for the following two reasons. First, the parallel analysis, a robust method for determining the optimal number of factors, in the EFA demonstrated that a one-factor structure was optimal. Second, the results of the inter-factor correlation in the CFA for two-factor models was very high at 0.926. Figure 2 shows the path diagram for the CFA of the J-IPAMP (the final one-factor model).

**Table 4:** Confirmatory factor analysis of the Japanese version of the instrument for patient assessment of medical professionalism

	**One-factor model**	**Two-factor model**
CFI	0.916	0.935
TLI	0.895	0.917
RMSEA	0.144	0.127
SRMR	0.045	0.044

*CFI* Comparative fit index, *TLI* Tucker–Lewis index, *RMSEA* Root mean square error of approximation, *SRMR* Standardized root mean square residual

To examine criterion-related validity, we calculated the Pearson correlation coefficients between the J-IPAMP total scores and the global rating. The Pearson correlation coefficient was 0.738, which exceeded the 0.30 criterion. The Cronbach’s alpha value of the 11 items was 0.96 (95% confidence interval: 0.96–0.97) and omega value was 0.96, indicating acceptable internal consistency reliability. Thus, we obtained the final version of the J-IPAMP (Table 5).

**Table 5:** Descriptive features of the Japanese version of the instrument for patient assessment of medical professionalism (*N* = 235)

Number of items	Mean	Median	Standard deviation	Observed range	Interquartile range	Skewness	Kurtosis
11	43.14	44.00	9.598	17.0–55.0	36.0–53.0	-0.473	-0.716

## Discussion

The English version of the IPAMP was translated into Japanese and examined in various aspects. The J-IPAMP showed good structural and criterion-related validity and internal consistency reliability. To the best of our knowledge, the J-IPAMP is the first validated scale that allows for the measuring of medical professionalism from the patient’s perspective in Japan.

This study has a number of methodological strengths. First, it followed a robust translation methodology. In keeping with international guidelines [21], we adapted the original English survey into Japanese using the following procedure: translation, synthesis, back translation, review by an expert in medical professionalism, and pilot testing. This robust methodology strengthened the validity of our findings. Second, the response rate was very high, at 85.1%. Although there is no clear definition of a high response rate, it is generally considered excellent when it exceeds 80% [33]. Obtaining a high response rate is essential for high-quality research that produces results with reliability, validity, and generalizability [33–35]. However, according to the National Research Council, response rates have continued to decline over time [36]. Research using patient-reported outcome measures has often encountered problems in recent years with low response rates owing to the lack of interest or time [37]. Our study achieved a high response rate, which diminished sampling bias concerns.

The original English version of the IPAMP has two factors (“involvement and respect” and “compassion and rapport”) [16], whereas an EFA of the J-IPAMP identified only one factor. This may have been the case for three reasons. First, there may have been a problem in the EFA of the original study, which concluded that EFA resulted in a two-factor structure despite the difference in factor loadings between the two factors having been small for all 11 items. It is possible that the original English version, similar to our Japanese version, had a one- rather than a two-factor structure. Second, there could be a translation issue. The original meaning of the instrument items may have changed slightly through the translation processes, which may have affected the respondents’ interpretation of the Japanese version. Third, cultural differences may have affected the results. Japan is generally considered a high-context culture [38], where behaviors and norms rely heavily on non-explicit rules and non-verbal cues that group members are expected to know implicitly [39]. In Japanese high-context communication, most information is withheld by the speaker, and little is in the explicit part of the message, whereas most information is vested in explicit expressions in low-context communication in the United States [40]. These cultural differences can lead to variations in expectations and assumptions for physician–patient communication. Ruhnke et al. noted that open discussions between doctors and patients are more limited in Japan than in the United States [41]. A comparative quantitative analysis of doctor–patient communication by Ohtaki et al. showed that there were differences between medical encounters in the United States and Japan in terms of interview time, proportion of time spent on various tasks, and use of back-channel responses [40]. Thus, the differences in physician–patient communication styles between both countries may have had an impact on the responses to the survey.

### Implications

This is the first Japanese version of the IPAMP. To the best of our knowledge, almost all previously developed tools for measuring physician professionalism were responded by healthcare professionals, whereas the IPAMP is responded by patients. In this era of patient-centered medicine, it is crucial to use the IPAMP, which incorporates the patient’s voice, in medical education. The J-IPAMP can be used to educate trainees in medical professionalism based on lived assessments by hospitalized patients in Japan. The development of versions in other languages would be worthwhile and welcome in order to promote international research.

### Limitations

This study had some limitations. First, the fit indices were not fully acceptable. A CFA should be conducted again in future studies when the data are fully accumulated. Further research to validate the fit indices would robust our tool, the J-IPAMP. Second, in this study, although the structural and criterion-related validity and internal consistency reliability of the scale were verified, we did not examine other psychometric properties (e.g., convergent and discriminant validity and test–retest reliability). Further validation of these properties can strengthen the developed tool. Third, the number of hospitals where the validation survey was conducted was relatively small. A larger multi-institute study would strengthen the instrument further. Fourth, as few studies have been found in terms of the assessment of physician professionalism by patients, it was difficult to compare our results with those of existing studies. However, this also underscores the novelty of the present study. Finally, we employed a global rating rather than selecting a specific scale to examine criterion-related validity.

## Conclusions

We translated the original English version of the IPAMP into Japanese, and then examined its structural and criterion-related validity and internal consistency reliability. Although further research is required for re-examining its structural validity, this instrument can be utilized for educating trainees on professionalism based on patients’ perspectives.

## Data Availability

The data sets generated and analyzed in this study are available from the corresponding author on reasonable request.

## Abbreviations

CFA: Confirmatory factor analysis
CFI: Comparative fit index
EFA: Exploratory factor analysis
IPAMP: Instrument for patient assessment of medical professionalism
KMO: Kaiser–Meyer–Olkin
RMSEA: Root mean square error of approximation
SRMR: Standardized root mean square residual
TLI: Tucker–Lewis index
